# Facets of Impulsivity in Relation to Diet Quality and Physical Activity in Adolescence

**DOI:** 10.3390/ijerph18020613

**Published:** 2021-01-12

**Authors:** Kathryn E. Smith, Jason M. Lavender, Adam M. Leventhal, Tyler B. Mason

**Affiliations:** 1 Department of Psychiatry and Behavioral Sciences, University of Southern California, Los Angeles, CA 90033, USA; 2 Department of Medicine, Uniformed Services University of the Health Sciences, Bethesda, MD 20814, USA; jason.lavender.ctr@usuhs.edu; 3 Military Cardiovascular Outcomes Research (MiCOR) Program, Bethesda, MD 20814, USA; 4 Metis Foundation, San Antonio, TX 78205, USA; 5 Department of Preventive Medicine, University of Southern California, Los Angeles, CA 90032, USA; adam.leventhal@usc.edu (A.M.L.); tylermas@usc.edu (T.B.M.)

**Keywords:** adolescents, diet, physical activity, impulsivity

## Abstract

Although the construct of impulsivity has generally been found to be associated with obesity and health behaviors in adults, research among adolescents is more limited and studies have yet to elucidate which facets of impulsivity may be most salient with regard to different eating and physical activity behaviors. Therefore, the present cross-sectional study assessed facets of impulsivity, measured by the UPPS-P questionnaire, in relation to health behaviors among adolescents. A sample of 2797 high school students from Los Angeles, California completed self-report measures during the ninth grade. The UPPS-P subscales (i.e., (lack of) premeditation, sensation seeking, (lack of) perseverance, negative urgency, positive urgency) were examined as predictors of unhealthy diet quality (i.e., frequency of consumption of high-fat foods and sweet food and drinks, measured by the National Cancer Institute (NCI) Quick Food Scan) and frequency of vigorous physical activity (measured by the Youth Risk Behavior Surveillance System). Greater sensation seeking, positive urgency, and negative urgency was associated with greater unhealthy diet quality (*p*s < 0.001). Greater negative urgency and lack of perseverance was associated with less frequent vigorous physical activity, whereas greater sensation seeking, lack of premeditation, and positive urgency was associated with more frequent vigorous physical activity (*p*s < 0.05). While negative urgency (i.e., impulsivity in the context of negative emotions) was consistently associated with poor health behaviors, other facets of impulsivity may potentiate vigorous physical activity in youth. Together these findings underscore the importance of considering the multidimensional nature of impulsivity in relation to adolescents’ health behaviors and highlight areas for future longitudinal research.

## 1. Introduction

Obesity is concerningly prevalent among youth, affecting approximately one in five children and adolescents in the United States [1,2]. Physical inactivity and poor diet quality represent key targets of pediatric obesity prevention and intervention [3], though effectiveness of behavioral and lifestyle interventions remains modest [4,5]. Moreover, adolescents show continual declines in physical activity and evidence poorer diet quality compared to other age groups [6,7]. In line with the socioecological model, these health behaviors are shaped at multiple levels of influence, which include intrapersonal (e.g., personality traits), interpersonal (e.g., family and peers), environmental (e.g., availability of foods and access to physical activities), and organizational factors (e.g., public policy) [8].

Prior research has indicated that impulsivity is a particularly relevant intrapersonal factor that is linked to obesity, and which may influence how youth engage with and navigate their social and built environments. Impulsivity is a multidimensional construct associated with risk-taking behaviors, difficulties maintaining attention, and poor planning ability [9]. Given the heterogeneity across theoretical models, definitions, and measures of impulsivity, the UPPS Impulsive Behavior Scale (and subsequently revised UPPS-P) 5-factor model of impulsivity may be useful to health behavior research [10,11]. Derived using data-driven methods (i.e., factor analysis of prior self-report measures), the UPPS-P measure assesses five distinct impulsivity facets: (1) lack of premeditation (i.e., the inability to delay action in favor of careful thinking and planning); (2) sensation seeking (i.e., the tendency to seek excitement and adventure); (3) lack of perseverance (i.e., the inability to remain with a task until completion and avoid boredom); (4) negative urgency (i.e., the tendency to act rashly when experiencing negative affect); and (5) positive urgency (i.e., the tendency to act rashly when experiencing positive affect [10,11]). 

Prior work has shown these facets of impulsivity are consistently associated with psychopathology and risky behavior [9], including among children and adolescents [12,13,14,15]. However, while impulsivity and self-regulation difficulties have been variously implicated in obesity and obesogenic behaviors among youth and adults [16,17], less work has examined associations between UPPS-P dimensions and energy balance health behaviors. Individuals with greater propensities for impulsivity across various facets may be more likely to display difficulties modulating the types and amount of food consumed, and/or experience difficulties implementing physical activity goals in the face of competing demands or more immediately reinforcing behaviors [18]. Further, this may be especially relevant for adolescents, who are be more likely to engage in reward-driven, sensation-seeking behaviors [19]. This is consistent with findings suggesting that adolescents with greater negative urgency report more frequent and energy-dense snacking [20], and that negative urgency (relative to other impulsivity facets) shows the strongest association with uncontrolled eating among young adolescents [21].

Together such data highlight adolescence as a key risk period for obesity development, and therefore examining personality-based correlates of energy balance behaviors may provide key insights for future obesity prevention and early intervention. In particular, these efforts may help to identify adolescents with health behavior profiles who are most at risk for developing excess weight, as well as elucidating potentially modifiable intervention targets. However, there is limited research in older youth examining associations between impulsivity facets and health behaviors. This is especially important given that adolescence is a time when youth have increasing autonomy over their eating behaviors and physical activity, and this is a critical period during which brain circuitry underlying executive functioning and self-control processes is continually developing [22]. Moreover, there is evidence that some facets of impulsivity (i.e., negative urgency) increase after puberty [9]. Thus, research applying multidimensional measures of impulsivity is crucial for understanding which facets may be most relevant to unhealthy patterns of dietary intake and physical activity behaviors among older adolescents. 

To address these gaps, the present study examined facets of impulsivity in relation to health behaviors among high school students from Los Angeles, California. Subscales of the UPPS-P questionnaire (i.e., (lack of) premeditation, sensation seeking, (lack of) perseverance, negative urgency, positive urgency) were examined simultaneously as predictors of unhealthy diet quality (i.e., frequency of consumption of high-fat foods and sweet food and drinks) and frequency of vigorous physical activity. It was hypothesized that higher levels of impulsivity would be negatively associated with unhealthy diet quality and physical activity. It was further hypothesized that negative urgency would emerge as a particularly salient impulsivity facet, although hypotheses regarding other specific facets were not made given the limited literature in this area. 

## 2. Method

### 2.1. Participants and Procedures

Participants were high school students in Los Angeles, California who took part in a longitudinal study of adolescent substance use and mental health. Approximately 40 schools were contacted, with a total of 10 schools recruited for the cohort survey [23]. The present study utilized assessments collected during the fall of 10th grade in 2014 (*N* surveyed = 3282, 96.6%); of these participants, 2797 had complete data for analyses (i.e., not missing UPPS-P, diet, or physical activity data). All students were eligible to participate, with the exception of those in special education or English as a second language programs. Participants and their parents provided assent and informed consent, respectively. Paper-and-pencil surveys were administered onsite in students’ classrooms. The study protocol was approved by the University of Southern California Institutional Review Board (#HS-12-00180).

### 2.2. Measures

#### 2.2.1. Demographics

Adolescents self-reported their gender, age, race/ethnicity, height, weight, and highest parental education from either parent (1 = 8th grade or less; 2 = Some high school; 3 = High school graduate; 4 = Some college; 5 = College graduate; 6 = Graduate degree). Weight and height were used to calculate body mass index (BMI; kg/m^2^). In addition to the measures reported in the current manuscript, the study included assessments of substance use and environmental and intrapersonal factors.

#### 2.2.2. Impulsivity

The UPPS-P Impulsive Behavior Scale (UPPS-P; [11]) measured lack of premeditation (e.g., “My thinking is usually careful and purposeful”), sensation seeking (e.g., “I’ll try anything once”), lack of perseverance (e.g., “I finish what I start”), negative urgency (e.g., “It is hard for me to resist acting on my feelings”), and positive urgency (e.g., “I tend to lose control when I am in a great mood”). Participants responded to items on a scale ranging from 1 (disagree strongly) to 4 (agree strongly), and relevant items were reverse scored and then averaged, such that higher subscale scores indicating greater impulsivity. The UPPS-P subscales demonstrate adequate psychometric properties [24], and Cronbach’s alphas for the subscales ranged from 0.87 to 0.96 in the current study.

#### 2.2.3. Diet Quality

The National Cancer Institute (NCI) Quick Food Scan was used to assess unhealthy dietary intake [25]. This measure has shown adequate validity when compared to 24-h dietary recall [26]. Food and drink items included sweet foods (i.e., candy, frozen desserts, and baked goods), high fat foods (i.e., hamburgers, fried chicken, tacos, pizza, and french fries), and sweet drinks (i.e., regular sodas, diet sodas, and sports drinks). Participants were asked to report how frequently they consumed foods and drinks items over the past 12 months on a scale ranging from 1 (never) to 8 (2 or more times per day). The NCI guidelines were used to transform each item into a daily consumption score by standardizing the midpoint of each frequency category to the number of times per day. For each of the three food categories, a maximum daily consumption score was computed and served as the indicator of dietary intake for that the respective food category; the average of these categories served as an index of unhealthy diet quality in the present study. 

#### 2.2.4. Youth Risk Behavior Surveillance System (YRBSS)

The YRBSS [27] is a validated measure of health behaviors in adolescents and was used in the present to assess the number of days in which participants engaged in vigorous exercise (i.e., “During the past week, how many days did you exercise or take part in physical activity that made your heart beat fast and made you breathe hard for at least 20 min?”). Responses were rated on an 8-point scale ranging from “None” (0) to “7 days” (7). 

### 2.3. Statistical Analyses

Descriptive statistics (mean and SD) and bivariate correlations (Pearson *r*) were calculated for study variables. A two-level multilevel linear mixed model was used to conduct analyses in order to account for non-independence of observations in the data (participants nested within schools) [28]. Multiple imputation was used to impute data for missing demographic covariates. Two separate multilevel models examined impulsivity facets as simultaneous predictors of each dependent variable (i.e., unhealthy diet quality, days of vigorous activity). Dependent variables were normally distributed and met assumptions for linear mixed models. Analyses controlled for demographic variables including gender, age, race, BMI, and highest parental education. Analyses were conducted using SPSS version 25.

## 3. Results

### 3.1. Descriptive Data

Table 1 shows descriptive statistics for the analyzed sample (mean age = 15.53 ± 0.42 years; mean BMI = 22.39 ± 4.51 kg/m^2^; 45.9% boys and 54.1% girls). The magnitude of intercorrelations among UPPS-P subscales ranged between 0.08 to 0.71. The racial/ethnic breakdown of the sample was as follows: 47.3% Hispanic, 16.0% White, 16.5% Asian, 6.6% Multiracial, 4.9% Black or African-American, 4.1% Native Hawaiian or Pacific Islander, 1.6% Other, and 1.0% American Indian or Alaskan Native (with 2.2% missing race/ethnicity data). With respect to parental education, 13.7% had less than eighth grade, 3.4% had some high school, 7.8% had high school, 14.5% had some college, 16.9% had a college degree, 27.3% had an advance degree, and 16.3% were unknown. 

Table 2 shows mixed model results. Covariates were not significantly related to diet quality, though vigorous physical activity frequency was lower among girls compared to boys (*B* = −0.51, *SE* = 0.09, *p* < 0.001), and among participants identifying as Native Hawaiian or Pacific Islander (relative to those identifying as White; *B* = −0.50, *SE* = 0.24, *p* = 0.041). Vigorous physical activity frequency was higher among participants whose parents had a higher level of education (*B* = 0.08, *SE* = 0.04, *p* = 0.035).

### 3.2. Unhealthy Diet Quality

After adjusting for covariates, sensation seeking, positive urgency, and negative urgency emerged as significant predictors of poor diet quality (sensation seeking: *B* = 0.11, *SE* = 0.03, *p* < 0.001; positive urgency: *B* = 0.16, *SE* = 0.04, *p* < 0.001; negative urgency: *B* = 0.15, *SE* = 0.04, *p* < 0.001), in that participants with higher scores in these domains reported poorer diet quality (Table 2). There were no significant associations of lack of perseverance or lack of premeditation with diet quality.

### 3.3. Days of Vigorous Physical Activity

All UPPS-P subscales were independently associated with frequency of vigorous physical activity days, though the direction of association varied across facets (Table 2). Participants with greater sensation seeking, lack of premeditation, and positive urgency reported a *higher* number of days on which they engaged in vigorous physical activity (sensation seeking: *B* = 0.51, *SE* = 0.06, *p* < 0.001; lack of premeditation: *B* = 0.27, *SE* = 0.08, *p* = 0.001; positive urgency: *B* = 0.20, *SE* = 0.09, *p* = 0.029). Conversely, participants with greater negative urgency and lack of perseverance reported a *lower* number of days on which they engaged in vigorous physical activity (negative urgency: *B* = −0.24, *SE* = 0.09, *p* = 0.005; lack of perseverance: *B* = −0.53, *SE* = 0.11, *p* < 0.001. 

## 4. Discussion

The present study examined facets of impulsivity in relation to health behaviors (i.e., diet quality and physical activity) among high school students. In line with expectations, sensation seeking and emotion-driven facets of impulsivity (i.e., positive and negative urgency) were independently associated with unhealthy dietary intake (i.e., frequency of consumption of high-fat foods and sweet food and drinks). A more complex pattern of findings emerged with respect to physical activity. That is, adolescents with greater negative urgency and lack of perseverance reported less frequent vigorous physical activity; conversely, those with greater sensation seeking, lack of premeditation, and positive urgency reported *more* frequent vigorous physical activity. 

### 4.1. Unhealthy Diet Quality

Results regarding diet quality were generally consistent with findings from adult samples demonstrating links between impulsivity and obesity-promoting eating behaviors, including intake of unhealthy foods and Western-style diets high in saturated fat and added sugar [29,30]. Importantly, the present findings add to and refine the growing literature in youth [31], and are consistent with results from prior research in children and adolescents showing associations between negative urgency, hedonic hunger, and dysregulated eating (e.g., emotional eating and binge eating) [32,33,34]. However, to our knowledge this is the first study in youth to demonstrate independent associations between positive urgency and diet quality, suggesting that relationships between urgency and some eating behaviors may not be specific to emotional valence (i.e., positive versus negative emotions). Adolescents high in urgency may consume palatable, high-calorie foods as a way of alleviating negative affect as well as in response to positive affect. In line with urgency theory, this may be due to increased reactivity to rewarding cues, such as palatable foods, coupled with diminished “top down” cognitive control brain circuitry [35]. Further, adolescents high in urgency may be particularly susceptible to these effects given that prefrontal brain regions that support self-regulation and modulation of reward are not fully developed in youth [36]. Thus, high levels of urgency may potentiate the likelihood of consuming rewarding, palatable foods, even in the face of alternative options. 

The observed relationship between sensation seeking and diet quality is also in line with prior research showing elevated sensation and novelty seeking among individuals with dysregulated eating behavior (i.e., binge eating) [37,38,39]. A recent study further showed differential relationships between dimensions of sensation seeking (i.e., experience seeking and boredom susceptibility) and binge eating among adolescent boys, although the pattern was not found in girls [40]. This highlights the importance of considering different sub-facets of sensation seeking in future research. In addition, assessing impulsivity facets in relation to both objective indices of diet quality and subjective reports of pathological eating (e.g., loss of control eating, binge eating) would be a helpful aim for future research, as it would help to delineate mechanisms underlying different types of maladaptive, eating behaviors.

Given the cross-sectional nature of the present findings, it is also possible that poor diet quality adversely impacts brain development and neurocognitive processes that support self-regulation and impulse control [41,42,43]. For instance, evidence suggests diet quality is positively associated with executive functioning, and consumption of diets high in fat and sugar may decrease impulse control by altering dopaminergic signaling. As such, further research is needed to examine directionality of the observed effects in this sample.

Of note, evidence has been equivocal regarding the extent to which impulse control difficulties are more generalized versus greater in the context of obesity or food-specific stimuli [44,45]. The present study used a generalized (i.e., non-food-specific) measure of impulsivity and findings emerged independent of weight status and gender, thus supporting general impulsivity as marker of obesity risk [31]. Nevertheless, further study is warranted to explore differences in effects across food-specific and generalized measures of impulsivity (e.g., Go/No-go paradigms with food and non-food stimuli), gender, and weight categories. 

### 4.2. Vigorous Physical Activity

Results regarding frequency of vigorous physical activity were more nuanced. The finding that adolescents with higher negative urgency and lack of perseverance reported less frequent vigorous physical activity was generally consistent with expectations. Given the broader literature on negative urgency and psychopathology and risky behaviors [9,12,13,14,15], adolescents with greater tendencies to act impulsively when experiencing negative affect may be more likely to resort to maladaptive behaviors as a way of coping with these aversive emotions. Additionally, youth who have more difficulty maintaining attentional focus or staying on task may be less likely to engage in sustained bouts of physical activity. Alternatively, it is possible that decreased physical activity adversely affects cognitive functioning that supports sustained attentional focus given that research among youth has shown higher physical activity is related to improved neurocognitive functioning [46]. Given that the present analyses were cross-sectional, future longitudinal research would be helpful to clarify the directionality of these associations.

In contrast to expectations, vigorous exercise occurred more frequently among adolescents with greater positive urgency, sensation seeking, and lack of premeditation. In line with prior literature in youth demonstrating positive affect improvement following increased physical activity [47], these results could suggest that these youth engage in vigorous physical activity as a way of increasing pleasant affective states, especially among those who have difficulties delaying behaviors. Further, youth high in sensation seeking may have enhanced sensitivity to pleasurable effects of physical activity and/or seek out intense physical activity activities as a form of stimulation, excitement, and adventure [19]. However, it is important to note that prior research also found that (negative) urgency and sensation-seeking were associated with problematic physical activity (i.e., exercise dependence or addiction [48]); therefore, in future research it will be important to differentiate between adaptive and maladaptive forms of exercise. 

### 4.3. Limitations

It is also important to note limitations of the present study. All analyses were cross-sectional and observational, which preclude inferences regarding temporal order of associations or causality. As noted, further longitudinal research is warranted to assess the direction of the observed effects, as well as explore the extent to which higher levels of physical activity may buffer prospective associations between impulsivity and unhealthy dietary intake during adolescence. Measures relied on retrospective reports of dietary intake and physical activity, and therefore future research could be enhanced via real-time data capture methods (e.g., ecological momentary assessment) and objective measures of physical activity (i.e., accelerometry). Further, the diet quality measure focused on unhealthy intake, and thus it is not clear how these facets of impulsivity may be correlated with intake of healthier foods (e.g., fruits and vegetables). Measures of disordered eating (e.g., loss of control eating, binge eating) and problematic exercise were also not administered; given known relationships between impulsivity and eating disorder symptoms, these measures would be useful to determine the extent to which the observed effects may differ based on presence of pathological eating and exercise behaviors. In addition, other dimensions of impulsivity (e.g., delay discounting) and or types of physical activity/inactivity (e.g., sedentary behavior) that were not assessed in the present study would be important to examine in research going forward. Finally, the present analyses focused on intrapersonal factors (i.e., impulsivity facets) without consideration of relevant contextual factors (e.g., people present, location, food environment) across levels of the socioecological model. These factors may mitigate and/or strengthen the observed associations between impulsivity and health behaviors, which would be helpful to explore in future studies using real-world, real-time data collection methods.

### 4.4. Conclusions and Future Directions

Despite these limitations, the current study nevertheless provides insight into differential associations between impulsivity facets and health behaviors during adolescence. Heightened levels of sensation seeking and emotion-driven facets of impulsivity (i.e., positive and negative urgency) emerged as factors linked to unhealthy dietary intake, while greater negative urgency and lack of perseverance were independently linked to less frequent vigorous physical activity. Contrary to expectations, greater sensation seeking, lack of premeditation, and positive urgency were related to *more* frequent vigorous physical activity, highlighting potential beneficial associations that warrant further consideration in physical activity promotion among youth.

In addition to the aforementioned future directions, the degree to which these associations may evolve during the transition to young adulthood and contribute to excess weight gain are important questions for further study. Given that facets of impulsivity may have distinct neural substrates [49], integration of neurobiological measures with longitudinal follow-ups may help to identify biobehavioral phenotypes of obesity risk during adolescence. Regarding clinical implications, the present results may inform interventions aiming to improve adolescents’ decision-making related to food intake and physical activity. Specifically, findings may help refine cognitive control training methods that target impulsivity-related behavior, though research in adolescents remains nascent [50,51]. Results regarding diet quality suggest that interventions should specifically focus on mindful decision-making and alternative sources of pleasure other than food, particularly when experiencing intense emotions. It also may be helpful to encourage physical activity as an alternative coping mechanism for youth with high emotion-driven impulsivity and/or stimulus-seeking traits. In summary, with ongoing research and longitudinal designs, these findings may provide insights into more targeted and timely obesity prevention and intervention for youth during critical risk periods. 

## Figures and Tables

**Table 1:** Descriptive statistics.

	*M*	*SD*
BMI	22.39	4.51
Age	15.53	0.42
Sensation seeking	2.28	0.75
Lack of perseverance	2.28	0.6
Lack of premeditation	2.25	0.75
Positive urgency	1.54	0.65
Negative urgency	1.97	0.70
Unhealthy diet quality ^1^	2.35	1.13
Vigorous physical activity days ^2^	3.64	2.37

Note. BMI = body mass index (kg/m^2^). ^1^ Mean unhealthy food and drink score (higher scores indicating poorer dietary quality) as measured by the National Cancer Institute (NCI) Quick Food Scan. ^2^ Days per week of vigorous physical activity as measured by the Youth Risk Behavior Surveillance System.

**Table 2:** Results from mixed models examining associations of impulsivity facets and covariates with unhealthy diet quality and vigorous physical activity frequency.

	Unhealthy Diet Quality (Mean Consumption Score ^2^)			Vigorous Physical Activity (Days per Week ^1^)		
	*B*	*SE*	*p*	*B*	*SE*	*p*
**Covariates**						
Race/ethnicity:						
Missing race/ethnicity data	0.29	0.16	0.071	−0.13	0.33	0.689
American Indian/Alaskan Native	−0.01	0.22	0.959	0.19	0.46	0.681
Asian	−0.15	0.08	0.057	−0.26	0.17	0.122
Black/African-American	0.1	0.12	0.370	−0.24	0.24	0.304
Hispanic/Latino	0.05	0.07	0.429	0.02	0.21	0.861
Native Hawaiian/Pacific Islander	0.08	0.12	0.487	**−0.50**	**0.24**	**0.041**
Other	0.01	0.1	0.906	−0.15	0.21	0.454
Multiracial	−0.06	0.18	0.722	−0.14	0.38	0.706
Gender	−0.07	0.04	0.129	**−0.51**	**0.09**	**<0.001**
Parental education	−0.03	0.02	0.100	**0.08**	**0.04**	**0.035**
BMI	−0.01	0.01	0.167	−0.01	0.01	0.334
Age	−0.01	0.04	0.766	0.04	0.09	0.627
**Impulsivity facets**						
Sensation seeking	**0.11**	**0.03**	**<0.001**	**0.51**	**0.06**	**<0.001**
Lack of perseverance	0.06	0.05	0.272	**−0.53**	**0.11**	**<0.001**
Lack of premeditation	−0.01	0.04	0.911	**0.27**	**0.08**	**0.001**
Positive urgency	**0.16**	**0.04**	**<0.001**	**0.20**	**0.09**	**0.029**
Negative urgency	**0.15**	**0.04**	**<0.001**	**−0.24**	**0.08**	**0.005**

Note. BMI = body mass index (kg/m^2^). Race was coded such that White was the reference category. Gender (male/female) was coded such that girls were the reference category. All independent variables were entered as simultaneous regressors. *B* reflects unstandardized estimates. Bold values indicate statistically significant effects (*p* < 0.05). ^1^ Days per week of vigorous physical activity as measured by the Youth Risk Behavior Surveillance System. ^2^ Mean unhealthy food and drink score (higher scores indicating poorer dietary quality) as measured by the National Cancer Institute (NCI) Quick Food Scan.

## Data Availability

The data presented in this study are available on request from the corresponding author.
